# Development and Optimization of a Thrombin Sandwich Aptamer Microarray

**DOI:** 10.3390/microarrays1020095

**Published:** 2012-08-08

**Authors:** Anna Meneghello, Alice Sosic, Agnese Antognoli, Erica Cretaio, Barbara Gatto

**Affiliations:** 1 Associazione CIVEN, Via delle Industrie 9, I-30175 Venezia-Marghera, Italy; Email: meneghello@civen.org (A.M.); cretaio@civen.org (E.C.); 2 Dipartimento di Scienze del Farmaco, University of Padova, Via F. Marzolo 5, I-35131 Padova, Italy; Email: alice.sosic@studenti.unipd.it; 3 Veneto Nanotech S.C.p.A., Via S. Crispino 106, I -35129 Padova, Italy; Email: agnese.antognoli@venetonanotech.it

**Keywords:** aptamer, thrombin, fluorescence, bioassay, multiplexing microarray

## Abstract

A sandwich microarray employing two distinct aptamers for human thrombin has been optimized for the detection of subnanomolar concentrations of the protein. The aptamer microarray demonstrates high specificity for thrombin, proving that a two-site binding assay with the TBA1 aptamer as capture layer and the TBA2 aptamer as detection layer can ensure great specificity at times and conditions compatible with standard routine analysis of biological samples. Aptamer microarray sensitivity was evaluated directly by fluorescent analysis employing Cy5-labeled TBA2 and indirectly by the use of TBA2-biotin followed by detection with fluorescent streptavidin. Sub-nanomolar LODs were reached in all cases and in the presence of serum, demonstrating that the optimized aptamer microarray can identify thrombin by a low-cost, sensitive and specific method.

## 1. Introduction

Thrombin, a serine protease involved in the last step of the coagulation cascade, produces insoluble fibrin through the proteolytic cleavage of soluble fibrinogen [1,2], and is characterized by two different exosites, one binding fibrinogen (fibrinogen binding domain, FBD) and the other binding heparin (heparin binding domain, HBD). The concentration of thrombin in blood varies considerably: it can be almost absent in the blood of healthy subjects but can reach low-micromolar concentrations during the coagulation process [3]. Thrombin, in addition to its direct actions on the coagulation system, has other functions as a potent signaling molecule that regulates physiologic and pathogenic responses: it represents, for example, a potent chemotactic agent for monocytes and leukocytes involved in the atherosclerotic and thrombotic processes [4]; it is a potential pro-inflammatory mediator in neurotrauma and neurodegenerative disorders [5] and it plays a role in the development, plasticity and pathology of the nervous system [6].

Considering thrombin’s physiological and pathological roles, it is not surprising that simple, low cost and sensitive methods for its detection are actively pursued, and human thrombin is one of the preferred targets for the development of aptamer-based assays. Aptamers are oligonucleotide ligands selected *in vitro* through the SELEX procedure (*Systematic Evolution of Ligands by EXponential enrichment*), and represent alternative options to antibodies for the development of diagnostics: besides their lower production costs, advantages of aptamers over antibodies are their relative ease of isolation and modification, tailored binding affinity and reversible denaturation, making them suitable candidates for use as detection systems [7,8,9]. A variety of aptamer-based protein detection strategies have been described in the past few years, employing in most cases two aptamers selected for targeting thrombin at its exosites: TBA1, a 15-nucleotide DNA aptamer able to bind FBD [10] and TBA2, a 29-nucleotide DNA aptamer able to recognize HBD [11]. These aptamers are known to assume, in presence of monovalent cations like K+ and Na+, a G-quadruplex structure [10,12,13]. Aptamer systems described in literature that employ both TBA1 and TBA2 present different formats and detection strategies including fluorescence, electrochemistry, inductively coupled plasma mass spectrometry (ICP-MS) and surface plasmon resonance (SPR), yielding variable limits of detection (LOD), ranging from nanomolar [14,15,16,17,18,19,20,21,22] to picomolar [23,24,25]. 

Among the aptamers detection platforms described, microarray represents an appropriate sensing format for high-throughput analysis, allowing to analyze a great number of samples at the same time and to scale up the system in order to obtain a multi-sensing platform [7]. In addition, a two-site binding approach is suitable for high-sensitivity detection of a target protein with two or more distinct target domains [26], allowing the setup of a sandwich on a solid support: this approach ensures higher assay specificity because the detection takes place only if the analyte is simultaneously recognized by two different ligands. After a thorough analysis of the ternary complex formation in solution we recently demonstrated the feasibility of the microarray strategy in a sandwich format for thrombin detection employing post-SELEX chemically modified TBA1 as capture layer and TBA2 as detection layer [27]. In the present work we describe the further development and optimization of the thrombin sandwich aptamer microarray for fluorescent analysis, evaluating the system specificity toward related and unrelated proteins. The microarray capability to detect thrombin was evaluated at different times and in complex biological matrices, and the limit of detection (LOD) and limit of quantification (LOQ) of the system were established. Finally, we compared the direct detection method employing the fluorescently labeled TBA2 with an indirect detection system using a biotin-labeled TBA2 subsequently recognized by fluorescent streptavidin. In both cases the aptamer arrays showed a sub-nanomolar detection limit even when tested in complex matrices containing serum, supporting the system capability to identify thrombin in a convenient, low-cost, sensitive and specific way amenable to multiplexing systems.

## 2. Materials and Methods

### 2.1. Aptamers and Proteins Solutions

The sequence of the unmodified 15-mer TBA1 [10], used here as selection aptamer, is: 5'-GGT TGG TGT GGT TGG-3'. To allow immobilization on microarray slides, an amino modification plus a polyT(12) spacer were added at the 5' terminus (TBA1(12T)NH_2_) as suggested by other authors [23]. In this way the G-quadruplex structure of TBA1, also if anchored to the slide surface, can fold correctly to recognize the target protein. The sequence of the unmodified 29-mer TBA2 [11], used here as selection aptamer, is: 5'-AGT CCG TGG TAG GGC AGG TTG GGG TGA CT-3'. TBA2 was used as selection aptamer with a 5'-Cy5 modification (TBA2-Cy5) or with a 5'-biotin modification (TBA2-biotin).

All protein molecules were purchased from Sigma-Aldrich (St. Louis, MO, USA): Human α-thrombin, Bovine Serum Albumin (BSA), Lysozyme, and Human Vascular Endothelial Growth Factor 165 (VEGF) were used to evaluate aptamer microarray specificity at a final concentration of 500 nM, and Cy3-labeled Streptavidin—at a final concentration of 30 nM—was used to perform thrombin assay in the presence of TBA2-biotin aptamer. Fetal Bovine Serum (FBS) (Gibco, Monza, Italy) was used in order to obtain a complex sample matrix.

### 2.2. Aptamer Preparation for Microarray Printing

In the aptamer arrays, chemically modified TBA1 is used as capture layer for thrombin [27]. Prior to immobilization on the microarray slide, TBA1(12T)NH_2_ (80 µM in the presence of KCl 100 mM) was denatured at 95 °C for 5 min and then left to cool down to room temperature. This folding step ensures molecules to assume a G-quadruplex structure, responsible for thrombin binding. Folded aptamer was then diluted in the Printing Buffer 1.5× (Printing Buffer 6×: 300 mM sodium phosphate, 0.02% Triton, pH 8.5) to a final concentration of 20 µM. TBA1 aptamers were loaded into microarray plates and submitted to the Microarray Spotter (Versarray Chip Writer Pro System, BioRad) for slide printing, using Telechem SMP3 microspotting pins (Arrayit, Sunnyvale, CA, USA).

### 2.3. Aptamer Microarray Printing

E-surf LifeLine slides (25 mm × 75 mm, LifeLineLab, Pomezia, Italy) were used for microarray printing: these slides allow the binding of amino modified oligonucleotides to the surface. On each slide up to 64 microarray sub-grids, made of six spots of capture aptamer were printed. Distance between spots centre was 200 μm and average spots diameter was 100 μm; distance between each sub-grid was 4.5 mm both in X and Y directions. Printed slides were incubated overnight in a 75% humidity incubation chamber (μBox, Quantifoil Instrument, Jena, Germany), blocked and stored according to protocol.

Each slide has the possibility to test up to 64 samples at once, since each sub-grid can be physically isolated from the others by multi-wells hybridization chamber (Grace Bio-Labs, OR, USA) during the incubation phase of the experiment. This approach ensures to analyze several samples on the same slide and nevertheless to minimize array variation resulting from minor fluctuation of external parameters.

### 2.4. Detection Layer Preparation

TBA2 (TBA2-Cy5 or TBA2-biotin) is used as secondary aptamer for detecting thrombin captured by the primary (capture) aptamer immobilized on the microarray. TBA2 (10 µM in KCl 100 mM) was denatured at 95 °C for 5 min and then left to cool down to room temperature in order to assume G-quadruplex structure, which is essential for recognition of the heparin binding domain of thrombin.

### 2.5. Sandwich Aptamer Microarray Assays

Printed aptamer microarrays prepared as detailed above were immersed, just before their use, in thrombin Binding Buffer 1× (20 mM Tris, 140 mM NaCl, 5 mM KCl, 1 mM MgCl_2_, pH 7.5) at room temperature for 30 min. Thrombin samples were pre-incubated with TBA2-Cy5 or TBA2-biotin in solution with thrombin Binding Buffer 1× (final volume 50 μL), at 25 °C for 30 min. The pre-formed complexes thus obtained were then incubated on the microarray at 25 °C for 2 h in the case of TBA2-Cy5. Steptavidin-Cy3 at the final concentration of 30 nM was added after 1 h of incubation in the case of TBA2-biotin, and the system allowed to incubating for 1 additional hour. Finally, aptamer microarrays were washed with PBS 1× (137 mM NaCl, 2.7 mM KCl, 10 mM Na_2_HPO_4_, 2 mM KH_2_PO_4_, pH 7.4) at room temperature to remove the unbound proteins and rapidly rinsed in MilliQ H_2_O.

### 2.6. Slides Scanning and Data Analysis

Spin-dried slides were scanned using GenePix 4000B laser scanner (Molecular Devices, Sunnyvale, CA, USA) and the GenePix Pro software using both 532 nm and 635 nm wavelength. Fluorescent spots intensities were quantified using the GenePix Pro software after normalizing the data by subtracting local background from the recorded spot intensities. For each set of six spots median and standard deviation were calculated. In calibration curves experiments LOD and LOQ were calculated: LOD is defined as 3 σ whereas LOQ is defined as 10 σ, where σ is the standard deviation of the blank experiment. All data analysis was performed with Origin Pro 8 software (OriginLab, Northampton, MA, USA).

## 3. Results and Discussion

### 3.1. Thrombin Specificity

We have developed an aptamer-based microarray exploiting two non-overlapping DNA aptamers recognizing different exosites of human thrombin [27]. Fluorescent labeling of the secondary aptamer TBA2-Cy5 allowed the co-localization of thrombin on TBA1 aptamer immobilized on microarray spots, thus proving the formation on a solid support of a sandwich in which the protein is simultaneously recognized by both aptamers. The recognition by aptamers was specific: a negative control represented by the aptamer OTA, selected for ochratoxin, was printed in the microarray to demonstrate the specific recognition of thrombin by TBA aptamers [27]: OTA aptamer was chosen because it can fold into a G-quadruplex after target binding [28], thus excluding non-selective recognition of G-quartets folded aptamers by our thrombin specific system.

With the aim of optimizing our system in view of future applications in multiplex microarray systems, we enlarged our analysis of the aptasensor specificity incubating the TBA1 microarray with proteins related and unrelated to human thrombin. Specificity toward unrelated proteins was evaluated using BSA and lysozyme [25], while human VEGF 165 was chosen for system specificity evaluation since it has both a fibrinogen [29] and a heparin binding domain (FBD and HBD respectively) like thrombin [30].

**Figure 1:**
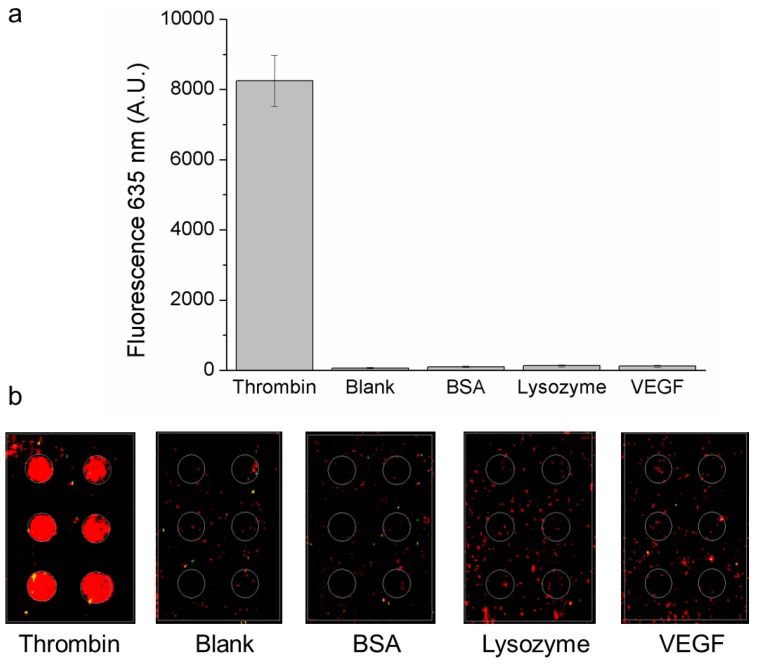
Specificity of aptasensor for thrombin. Fluorescence signal (635 nm) recorded on thrombin aptamer microarray after the incubation of the different indicated proteins with TBA2-Cy5 (protein and TBA2 final concentration: 500 nM and 1 mM, respectively); (**a**) thrombin, blank (TBA2-Cy5 only), BSA, lysozyme and VEGF and (**b**) corresponding microarray images.

Figure 1 shows that no significant fluorescent signal was recorded on TBA1 microarray spots after incubation of samples containing BSA or lysozyme pre-incubated with TBA2-Cy5, confirming the specificity of the two aptamers towards human thrombin. The aptasensor is able to discriminate between related target domains: experimental data in Figure 1 shows clearly that no significant binding on TBA1 spots was observed in presence of VEGF 165.

These results hence confirm that TBA1 and TBA2 used in the two-site binding assay ensure high recognition specificity and are able to discriminate between HBD and FBD from different proteins.

### 3.2. Effect of Incubation Time

**Figure 2:**
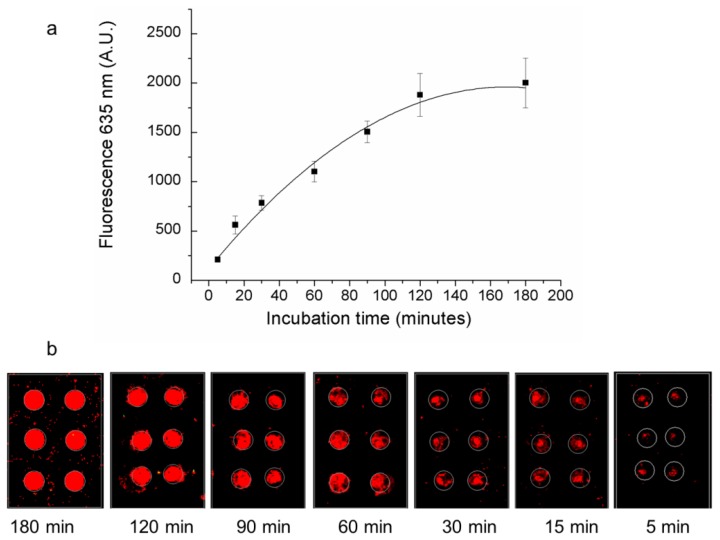
Effect of incubation time. Fluorescence signal (635 nm) recorded on TBA1 microarray (**a**) after the incubation of thrombin and TBA2-Cy5 for different times (180–120–90–60–30–15–5 min) and (**b**) corresponding microarray images.

Assay incubation time was optimized in order to obtain the maximum fluorescent signal from analyzed samples and at the same time to perform the assay within a time compatible with routine analysis. After a pre-incubation step between the protein and the detection aptamer TBA2-Cy5, samples were incubated on the TBA1-printed microarray slide for a time ranging from 5 min up to 180 min. As shown in Figure 2 the fluorescent signal from TBA2-Cy5 increases exponentially over time, reaching a maximum intensity after 180 min, but the difference between the relative fluorescence intensity at 2 h and 3 h incubation time is lower than 6%. To achieve high assay sensitivity and to perform the assay in an overall time compatible with that of standard commercial assays, we performed all the following experiments using a 2 h incubation time.

### 3.3. Effect of Serum

**Figure 3:**
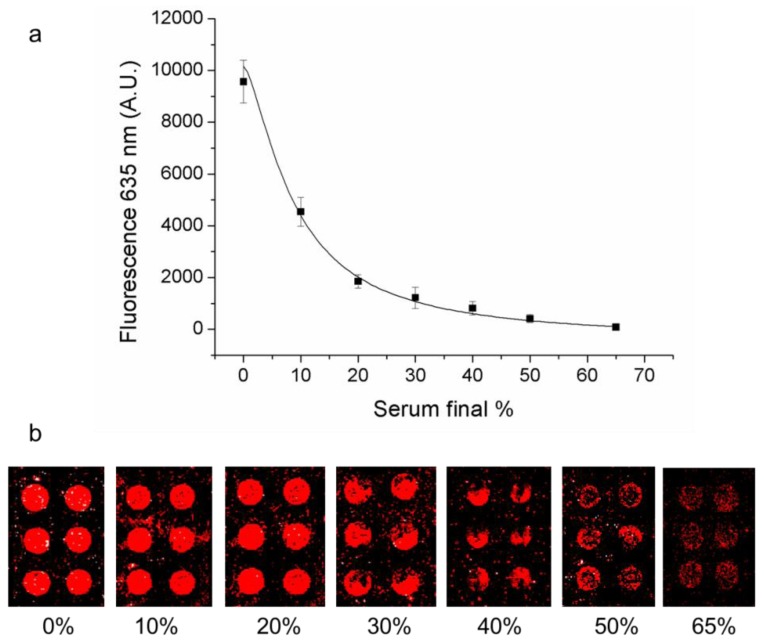
Effect of serum. Fluorescence signal (635 nm) recorded on TBA1 microarray (**a**) after the incubation of thrombin and TBA2-Cy5 complexes in presence of different Fetal Bovine Serum (FBS) dilutions (0%–10%–20%–30%–40%–50%–65%) and (**b**) corresponding microarray images.

Immunoassays protocols require sample dilutions prior to analysis in order to dilute possible interferents [31]. To evaluate the system sensing ability in complex matrixes, equal thrombin amounts (500 nM) were analyzed in the presence of fetal bovine serum (FBS) at different dilutions ranging from 0% to 65%. After a short pre-incubation in the presence of FBS, samples were incubated for two hours on the array slide printed with TBA1. Collected data shown in Figure 3 demonstrate that the system can accurately work in the presence of 20% as well as 10% FBS without affecting the system performance. A 20% FBS solution, corresponding to a biological sample dilution of 1:5 is therefore compatible with the sandwich aptamer microarray analysis. 

### 3.4. Limit of Detection of Direct and Indirect Methods

**Figure 4:**
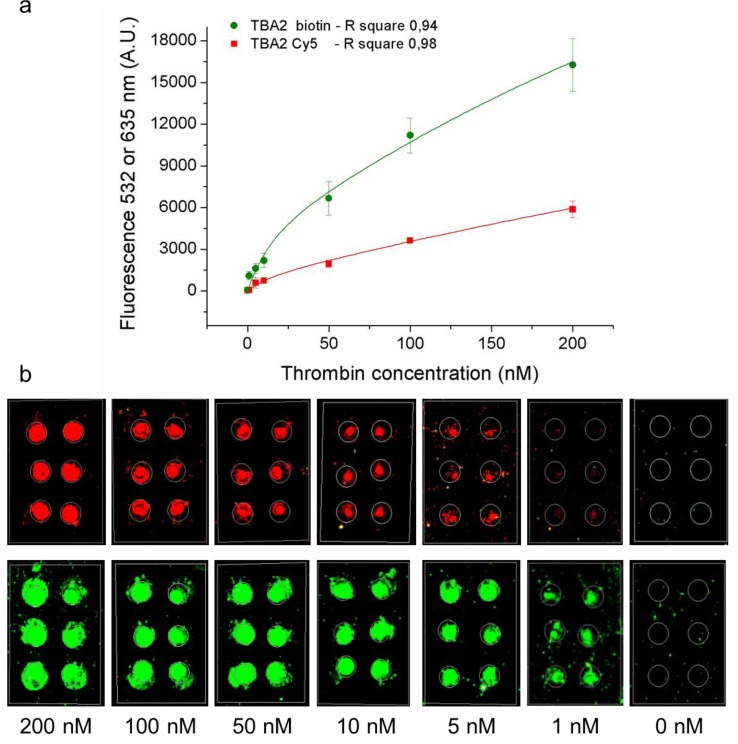
Direct and indirect method for thrombin detection. (**a**) Fluorescence signal (635 or 532 nm) plot of thrombin aptamer microarray after the incubation of thrombin and TBA2-Cy5 (direct method, red squares, 635 nm) or thrombin and TBA2-biotin plus Cy3-streptavidin (indirect method, green dots, 532 nm) and (**b**) relative microarray images. Tested thrombin dilutions were: 200 nM–100 nM–50 nM–10 nM–5 nM–1 nM–0 nM. TBA2-Cy5 (red squares) or TBA2-biotin (green dots) concentration was 400 nM. For the indirect method the microarray slide was incubated for 1 h with Cy3-streptavidin, for a total incubation time of two hours in all cases.

The microarray developed and described is based on the “direct” detection of the labeled secondary aptamer recognizing the analyte-aptamer complex on the slide. This system was compared to an “indirect” method based on the formation of a similar sandwich-type on the microarray, but employing a secondary aptamer conjugated with biotin, followed by a detection step with fluorescently labeled streptavidin. Cy3-streptavidin has 3–9 fluorophores (Cy3) linked per molecule of protein: thus, the use of TBA2 biotin plus Cy3-streptavidin could potentially allow amplification of the signal reaching lower LOD and LOQ. This indirect method would also allow the development of a multiplex systems employing biotinylated aptamers specific for each analyte followed by a single detection step with labeled streptavidin. We set up and compared both methods for the thrombin microarray, and evaluated the assay limit of detection (LOD) and limit of quantification (LOQ) in both cases: different thrombin concentrations (200 nM–100 nM–50 nM–10 nM–5 nM–1 nM–0 nM) were pre-incubated with the two different secondary aptamers (respectively TBA2-Cy5 and TBA2-biotin) and finally laid on the TBA1 microarray slide. In the first (direct) case, the microarray is analyzed at 635 nm fluorescent excitation to detect Cy5 directly conjugated to the aptamer, in the second (“indirect”) method the microarray is analyzed at 532 nm fluorescent excitation to detect Cy3-labeled streptavidin laid over the treated samples. For each dilution, the mean (subtracted to background values) of six spots was calculated, with corresponding standard deviation. As shown in Figure 4, the direct method (red dots) employing TBA2-Cy5 yields a LOD of 0.75 nM and LOQ of 2.85 nM, corresponding to 27 ng/mL and 102.6 ng/mL of thrombin, respectively. In the indirect method with TBA2-biotin detected by fluorescent streptavidin (green dots), a LOD of 0.17 nM and LOQ of 0.97 nM were obtained, corresponding to 6.12 ng/mL and 34.92 ng/mL of thrombin, respectively. The indirect method allows therefore to obtain a more sensitive detection system and has been applied to detect thrombin in presence of FBS, as shown in the following Paragraph.

### 3.5. Indirect Method for Aptasensor Detection: Effect of Serum

**Figure 5:**
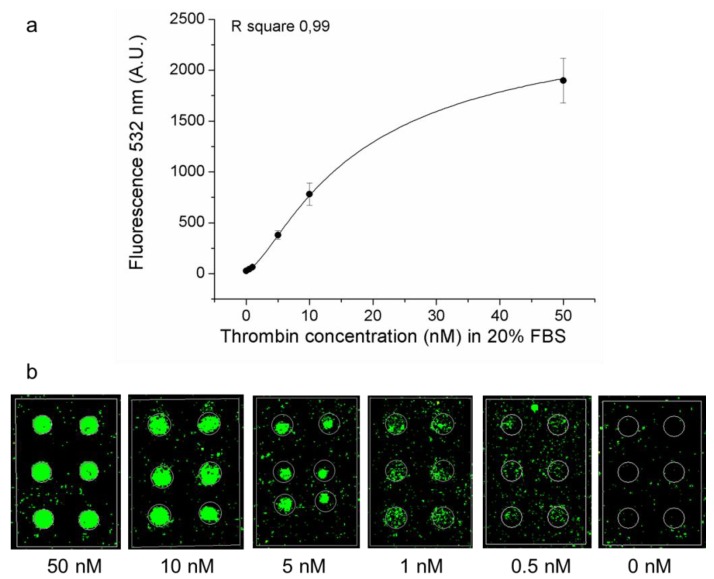
Thrombin calibration curves in FBS solution. (**a**) fluorescence signal (532 nm) plot of TBA1 microarray in 20% FBS solution after the incubation of thrombin and TBA2-biotin plus Cy3-streptavidin and (**b**) corresponding microarray images. Tested thrombin dilutions were: 50 nM–10 nM–5 nM–1 nM–0.5 nM–0 nM, with a fixed TBA-2 biotin concentration of 200 nM.

To complete our characterization of the aptamer microarray for thrombin detection based on the indirect method of fluorescent analysis, samples with decreasing concentrations of thrombin were analyzed in solutions containing serum, in order to evaluate the sensing ability to detect thrombin in a complex matrix. Samples with decreasing concentrations of thrombin (50 nM–10 nM–5 nM–1 nM–0.5 nM–0 nM) and 20% FBS, were pre-incubated in presence of 200 nM of TBA2-biotin and finally incubated on the microarray slide as previously described. After Cy3-labeled streptavidin incubation, data were collected and reported in Figure 5. The presence of 20% serum contributes to lower the overall specific detection signal, as expected, but the LOD and LOQ, respectively 0.25 nM and 1.26 nM (corresponding to a thrombin concentration of 9 ng/mL and 45.36 ng/mL, respectively) allows sub-nanomolar detection of the analyte also in these conditions.

## 4. Conclusions

Analytical approaches based on biosensors for protein recognition and quantification have extensively been analyzed in the past few years [32,33]. The identification of disease-related biomolecules in body fluids is useful for the diagnosis of diseases as well as for follow-up protocols in clinical settings. Analytes can be identified with different transducing and recognition strategies, including antibodies, enzymes or aptamers: each different approach needs to be optimized in order to achieve increasing selectivity and specificity, and reduced assay time and costs. The data shown here demonstrate that the microarray based on aptamer recognition of an analyte is efficient and specific, and different detection aptamers and methods can be utilized. We have shown that the use of the indirect method (TBA2-biotin plus Cy3-streptavidin) allows lower LOD and LOQ when compared to TBA2-Cy5 (direct method) even in the presence of biological fluids. Moreover, the signal amplification made possible by the use of TBA2-biotin plus Cy3-streptavidin as detection strategy allows for a stronger signal at low protein concentrations, as shown in Figure 4. 

The TBA1 microarray exhibits a limit of detection comparable or superior to other systems described in the literature employing the same aptamers and different technologies [17,18,34] but has the advantage of a simple set up that could be exploited for the development of a multiplex microarray for protein and genome analysis in presence of a single detection strategy. Besides, the aptasensor could be further developed with signal amplification strategies by the use of fluorescent nanoparticles conjugated with streptavidin to allow for nanotech systems, to reach lower detection limits comparable to those achieved by different assay formats or technologies [23,25,35].
